# Treatment outcomes of patients with MDR-TB and its determinants at referral hospitals in Ethiopia

**DOI:** 10.1371/journal.pone.0262318

**Published:** 2022-02-17

**Authors:** Mengistu K. Wakjira, Peter T. Sandy, A. H. Mavhandu-Mudzusi

**Affiliations:** 1 Abt Associates Inc Ethiopia, Addis Ababa, Ethiopia; 2 Buckinghamshire New University, Uxbridge Campus, Uxbridge, London, United Kingdom; 3 University of South Africa, College of Human Sciences, Office of Graduate Studies and Research, Pretoria, South Africa; Institute of Medical Sciences, Banaras Hindu University, INDIA

## Abstract

**Background:**

There is limited empirical evidence in Ethiopia on the determinants of treatment outcomes of patients with multidrug-resistant tuberculosis (MDR-TB) who were enrolled to second-line anti-tuberculosis drugs. Thus, this study investigated the determinants of treatment outcomes in patients with MDR-TB at referral hospitals in Ethiopia.

**Design and methods:**

This study was underpinned by a cross-sectional quantitative research design that guided both data collection and analysis. Data is collected using structured questionnaire and data analyses was performed using the Statistical Package for Social Sciences. Multi-variable logistic regression was used to control for confounders in determining the association between treatment outcomes of patients with MDR-TB and selected predictor variables, such as co-morbidity with MDR-TB and body mass index.

**Results:**

From the total of 136 patients with MDR-TB included in this study, 31% had some co-morbidity with MDR-TB at baseline, and 64% of the patients had a body mass index of less than 18.5 kg/m^2^. At 24 months after commencing treatment, 76 (69%), n = 110), of the patients had successfully completed treatment, while 30 (27%) died of the disease. The odds of death was significantly higher among patients with low body mass index (AOR = 2.734, 95% CI: 1.01–7.395; P<0.048) and those with some co-morbidity at baseline (AOR = 4.260, 95%CI: 1.607–11.29; p<0.004).

**Conclusion:**

The higher proportion of mortality among patients treated for MDR-TB at Adama and Nekemte Hospitals, central Ethiopia, is attributable to co-morbidities with MDR-TB, including HIV/AIDS and malnutrition. Improving socio-economic and nutritional support and provision of integrated care for MDR-TB and HIV/AIDS is recommended to mitigate the higher level of death among patients treated for MDR-TB.

## Background

During the past two centuries, tuberculosis (TB) has claimed more lives than any other infectious disease on earth [1]. Despite advances in the treatment, prevention and control of the disease, tuberculosis continues to be one of the major causes of morbidity and mortality worldwide [2–5]. This is attributed mainly to the global rise in the incidence of MDR-TB [6–8]. MDR-TB limits treatment options for the disease. Treatment of MDR-TB can take up to two years with drugs that are poorly tolerated and difficult to monitor. Moreover, the treatment outcomes of patients with MDR-TB are generally poor [9–17]. Several factors have an impact on the outcomes of the treatment given for MDR-TB. Social and financial hardships, including undernutrition, enhance vulnerability to tuberculosis and challenge the process and the outcomes of the treatment given for the disease [18–22]. Co-morbidities with MDR-TB, including HIV, malignancies, diabetes mellitus and chronic renal failure are risk factors for death among patients with MDR-TB [23–31]. Moreover, adverse drug reactions from second-line drugs and MDR-TB associated stigma have a negative impact on the outcomes of the treatment given for MDR-TB [32, 33]. Without addressing these factors, the mere provision of free drugs may not directly lead to optimum treatment outcomes of patients treated for the disease [34, 35].

Ethiopia is one of the global high-burden countries for TB, TB/HIV and MDR-TB [16]. Yet, the number of MDR-TB cases detected and enrolled for treatment in the country has been far below the national incident estimate [17]. The huge pool of untreated cases of MDR-TB represents an important source of disease transmission [19]. Thus, the government of Ethiopia is expanding the services on the programmatic management of MDR-TB to all its regions using community-based ambulatory model of care [20].

In Ethiopia, there is very limited empirical evidence on factors determining the outcomes of the treatment given for patients with MDR-TB based on the community-based ambulatory model of care. Absence of such empirical evidence is a challenge to understand the desired impact of the national MDR-TB service expansion. Thus, we undertook a study to assess the determinants of the treatment outcomes of patients with MDR-TB at two referral hospitals found in the Oromia region of Ethiopia.

### Aim and objectives

This study aimed to investigate the treatment outcomes of patients with MDR-TB and its determinants at referral hospitals in the Oromia region of Ethiopia. The specific objectives of the study were to:

- determine the treatment outcomes of patients with MDR-TB who are enrolled on second-line anti-tuberculosis drugs.
- assess factors associated with observed levels of treatment outcomes among patients treated for MDR-TB.

## Materials and methods

### Study design

The study utilised a cross-sectional quantitative research design to assess, describe and analyse the treatment outcomes of patients with MDR-TB, and its determinants. Thus, a deductive approach was used to test the plausible relationship between the treatment outcomes of patients with MDR-TB and its determinants.

### Study setting and sampling

The study was conducted at Adama and Nekemte Referral Hospitals in the Oromia region of Ethiopia. In the Oromia region of Ethiopia, the programmatic management of MDR-TB was first initiated at these hospitals. Thus, it was assumed that the two hospitals had adequate experience and data on the programmatic management of MDR-TB. The population of the study consisted of all patients with laboratory-confirmed MDR/RR-TB enrolled for the treatment for MDR-TB at the two hospitals. Between 26 December 2012 and 17 September 2016, a total of 182 patients with MDR-TB were enrolled for treatment at the two hospitals. Data is collected from all patients who were on treatment for a period of six months or above at the time of data collection. 46 (25%) patients did not meet the inclusion criteria (less than 6 months on treatment by the time of data collection and for whom at least interim treatment outcome could not be determined) and excluded from the study. Thus, data is collected from the remaining 136 (75%) patients that fulfilled the inclusion criteria. All patients that met the inclusion criteria were included in the study to make sure that sufficient number of the required sample size is included in the study and inferences could be made. The main variable of interest among the clinical characteristics of patients with MDR-TB was the presence of co-morbidity with MDR-TB. There are two groups with respect to this: group of patients with co-morbidity (p1) and groups without co-morbidity (p2) with MDR-TB at the baseline. From the literature P1 & P2 for this study are, P1 = 0.81 and p2 = 0.70 [39]. The calculated power for the sample size included in this study is 0.87. Thus, clinical record of all the 136 participants who meet the inclusion criteria is retrieved and included in the study.

### Data collection and analysis

Ethical approval to conduct the study was obtained from the Department of Health Studies of the University of South Africa (UNISA) and from Oromia Region Health Bureau, Department of Public Health Emergency Management and Health Research. To get access to patients’ medical records at each study site, informed consent was sought and obtained from hospital management and clinical caregivers [36]. The study was conducted between 10 November 2016 and 7 February 2017, by using a structured questionnaire. Study variables were extracted from extant literature and conceptualised and operationalised in line with the aims and objectives of the study. The questionnaire was serially reviewed by experienced experts and subjected to preliminary field testing and further amendment before its use on the main study. Data was collected by the principal investigator and two healthcare professionals who were offered two days’ training on the specifics of data collection for this study. Data is analysed using Statistical Package for Social Sciences [37]. Categorical variables were summarised as frequencies and percentages. A bivariate analysis was performed to identify factors associated with the treatment outcome of patients with MDR-TB. A multi-variable logistic regression analysis was employed to determine the independent predictors of the treatment outcomes of patients with MDR-TB. The results of the logistic regression are expressed as crude and adjusted odds ratio. Confidence intervals and p-values were used to test significance of the observed sample parameters in exploring determinants of the treatment outcomes of patients treated for MDR-TB.

## Results

### General characteristics of the participants

A total of 136 patients (n = 136) with MDR-TB were included in the study. 74 (54%) were male and 62 (46%) were female patients with MDR-TB. Altogether, 128 (94%) of the patients were in the productive age group of 15 to 64 years. Moreover, 28 out of 30 (93%) of the total deaths from MDR-TB occurred in the same age group. 4 (3%) of the patients were aged less than 15 years, while 4 (3%) were aged 65 years and above. The mean age of the study participants (mean ± SD) was 32.12 ± 12.53 while the age range of the participants was 4 to 73 years. From those within age of employment (n = 132), 70 (53%) were self-employed mainly in the informal labour work while 46 (35%) were not employed (Table 1).

**Table 1:** Socio-demographic and clinical characteristic of study participants (n = 136).

Parameter	n (%)
Sex (n = 136)	
Male	73 (54)
Female	63 (46)
**Age category (n = 136)**	
<15 years	4 (3)
15–44 years	110 (81)
45–64 years	18 (13)
>/ = 65 years	4 (3)
**Patients’ employment status (n = 132)**	
Formally employed	7 (5.3)
Self-employed	70 (53)
Unemployed	46 (35)
Other	9 (7)
**Patients’ drug-resistance type at diagnosis (n = 136)**	
RR	89 (65)
MDR-TB	47 (35)
**HIV test result (n = 131)**	
HIV positive	34(26)
HIV negative	97 (74)
**Presence of co-morbidity at baseline (n = 133**)	
Yes	41 (31)
No	92 (69)
**Type of co-morbidity at baseline (n = 41)**	
HIV/AIDS	34 (83)
Diabetes mellitus	5 (12)
Other	2 (5)
**Body mass index (BMI) (n = 136)**	
BMI <18.5Kg/m^2^	87 (64)
BMI >/ = 18.5Kg/m^2^	49 (36)
**Site of the TB disease (n = 136)**	
Pulmonary	134 (98)
Extra-pulmonary	2 (2)
**Type of the TB case (n = 136)**	
Bacteriologically confirmed PTB	134 (98)
Bacteriologically confirmed EPTB	1 (1)
Clinically diagnosed EPTB	1 (1)
**Result of diagnostic sputum smear examination (n = 132)**	
Smear positive	105 (79)
Smear negative	27 (21)
**Sputum bacillary load reported at diagnosis (n = 132)**	
No AFB seen	27(20)
Scanty	59(45)
Moderate	41(31)
High	5(4)

n = number; % = percent.

### Clinical characteristics of the participants

Altogether 134 (98%) of the patients were bacteriologically confirmed pulmonary MDR-TB cases, while 2 (2%) were bacteriologically confirmed extra-pulmonary MDR-TB. 90 (66%) of the patients were diagnosed with MDR-TB after failure of re-treatment regimen with first-line anti-TB drugs, while 17 (13%) were diagnosed after failure of the standard six-month regimen with first-line anti-tuberculosis drugs. A total of 14 (10%) patients were registered for treatment after relapse, while 11 (8%) were new cases of MDR-TB without any prior history of treatment with anti-tuberculosis drugs. 4 (3%) of the patients were diagnosed among patients returning after lost to follow-ups. From patients with documented baseline sputum microscopy test (n = 132), 105 (79%) were sputum smear positive and 27 (21%) were sputum smear negative. An analysis of initial bacillary load (n = 132) revealed that the initial bacillary load for 59 (45%) patients was scanty, and it was moderate for 41 (31%) and high for 5 (4%) patients. Altogether 89 (65%) of the total patients had a drug-susceptibility test result by GeneXpert and they were resistant to rifampicin. Only 47 (35%) of the total patients were diagnosed using either culture or line probe assay tests. This group of patients had drug-susceptibility test result for both rifampicin and isoniazid, and they were resistant to both drugs. No patient had a drug-susceptibility test result for any of the second-line anti-tuberculosis drugs. A total of 41 (31%) patients (n = 133) had some co-morbidity with MDR-TB at baseline, out of which 34 (83%) was due to co-infection with HIV. 5 (12%) patients had diabetes mellitus and each of 2 (5%) patients had cardio-vascular and kidney diseases. 87 (64%) of the 136 patients had body mass index (BMI) of less than 18.5kg/m^2^ at baseline, which is indicative of malnutrition as co-morbidity with MDR-TB (Table 1). Complete data on adverse drug reactions from second-line anti-TB drugs was retrieved only for 91 (67%) (n = 91), of patients included in the study, and all these patients experienced at least one episode of adverse drug reactions in the course of their treatment. A total of 31 (34%) patients experienced five or more episodes of adverse drug reactions, 12 (13%) experienced four episodes, 14 (15%) experienced three episodes, 22 (24%) experienced two episodes, while the remaining 12 (13%) experienced one episode of adverse drug reactions. By the body organs involved, 73 (80.2%) patients developed gastro-intestinal tract-related adverse reactions and 35 (38.5%) patients had neurological-related adverse drug reactions. Musculoskeletal-related adverse reactions occurred among 26 (28.6%), followed by cardio-vascular-related adverse drug reactions that occurred in 24 (26.4%) patients. Electrolyte disturbances occurred among 13 (14.3%) patients and 11 (12%) developed psychiatric-related adverse drug reactions. Moreover, 5 (5.5%) of the patients developed adverse drug reaction involving the eye while 3 (3.2%) developed immune related adverse drug reactions. Permanent loss of hearing occurred in 7 (7.7%) patients and 1 (1%) patient died of suicide while on treatment. Analysis of trend of occurrence of the adverse drug-reactions revealed that except for musculo-skeletal and neurological-related adverse drug reactions, most of the adverse drug reactions occurred during the first six to eight intensive-phase months of patient treatment (Table 1).

### Treatment outcomes of patients with MDR-TB

At six months of follow-ups, 98 (72%) of the patients were culture negative and 26 (19%) died of the disease while the six month’s interim treatment outcome was not evaluated and documented for 12 (9%) of the patients (Table 2).

**Table 2:** The treatment outcomes of patients with MDR-TB at six and 24 months after commencing treatment.

Six-month treatment outcomes (n = 136)		24-month (final) treatment outcomes (n = 110)	
Parameter	n (%)	Parameter	n (%)
Culture negative	98 (72)	Cured	65 (59)
Culture positive	0 (0)	Treatment completed	11 (10)
Died by sixth month	26 (19)	Composite treatment success rate	76 (69)
		Died by 24 months	30 (27)
Not evaluated	12 (9)	Lost to follow-ups	1 (1)
Lost to follow-ups	0 (0)	Not evaluated	3 (3)
		On treatment	26 (19)

n = number; % = percent.

The 24 months’ treatment outcome was determined for 110 (81%) of the patients. At 24 months, 76 (69%) had successfully completed their treatment. Thus, the composite treatment success rate for patients included in this study was 69%. Altogether 30 (27%) patients died of the disease at the 24 months’ follow up period. The treatment outcomes of 3 (3%) patients were not evaluated at month 24, mainly due to patient transfers to other treatment centres and reports on their treatment outcomes were not available by the time of data collection. 1 (1%) patient was lost to follow-ups and not retrieved by the time of data collection.

### Determinants of the treatment outcomes of patients with MDR-TB

Bivariate analysis revealed that from the total of 30 deaths that occurred among all patients, 19 (42%) was among female patients, while 11 (23%) was among male patients. Compared to male patients with MDR-TB, the odds of death was higher among female patients with MDR-TB (Crude OR = 2.436; X^2^ = 4.459; P<0.035; 95%CI = 1.066–5.566). Compared to patients without any co-morbidity at the baseline, the odds of death was higher among patients with some co-morbidity with MDR-TB at the baseline (Crude OR = 2.864; X^2^ = 5.802; P<0.016; 95%CI = 1.217–6.743). Likewise, compared to HIV-negative patients with MDR-TB, the odds of death was higher among patients co-infected with HIV (Crude OR = 2.741; X^2^ = 4.795; P<0.029; 95%CI = 1.112–6.761). Moreover, compared to patients whose body mass index (BMI) was greater than or equal to 18.5kg/m^2^, the odds of death from MDR-TB was higher among patients with BMI <18.5kg/m^2^_._ (Crude OR = 2.925; X^2^ = 5.327; P<0.021; 95%CI = 1.176–7.277) (Table 3).

**Table 3:** Summary of bivariate analysis on the determinants of MDR-TB treatment outcomes of the study participants at Adama and Nekemte Hospitals, Oromia, Ethiopia, December 2012 –September 2016 (n = 110).

Variable	Category	Favourable treatment outcome (cured or treatment completed) n (%)	Unfavourable treatment outcome (Death) n (%)	Total	Crude OR^@^	Wald x^2^ test result	P-value	95% CI^@@^
				n (%)				
Sex	Male	54 (77)	11 (23)	65 (59)	____	_____	____	______
	Female	26 (58)	19 (42)	45 (41)	2.436	4.459	<0.035	1.066–5.566
BMI	>18.5Kg/m^2^	36 (82)	8 (18)	44 (40)	____	_____	____	______
	</ = 18.5Kg/m^2^	40 (60)	26 (39)	66 (60)	2.925	5.327	<0.021	1.176–7.277
Any co-morbidity at baseline	No	58 (76)	18 (24)	76 (69)	____	_____	____	______
	Yes	18 (53)	16 (47)	34 (31)	2.864	5.802	<0.016	1.217–6.743
HIV	Negative	62 (75)	21 (25)	83 (75)	____	_____	____	______
	Positive	14 (52)	13 (48)	27 (25)	2.741	4.795	<0.029	1.112–6.761
Resistance type	RR-TB	39 (61)	25 (39)	64 (58)	____	_____	____	______
	MDR-TB	37 (80)	9 (20)	46 (42)	2.635	4.608	<0.032	1.088–6.384

n = number; % = percent

@ = Odds Ratio

@@ = Confidence Interval.

Furthermore, the presence of fibrotic (extensive) lung lesion, which is indicative of advanced disease status, was revealed to be predictor of death from MDR-TB (Phi X^2^ = 0.405, P<0.017).

The final multivariable logistic regression analysis (Table 4) revealed that the odds of death among patients with MDR-TB who had some co-morbidity with MDR-TB at the baseline was significantly higher than the odds of death among those without co-morbidity (AOR = 4.260, 95%CI: 1.607–11.297; P<0.004). Moreover, the odds of death from MDR-TB among patients with low body mass index (MBI) was 2.7 times higher than the odds of death among patients with body mass index greater than or equal to 18.5kg/m^2^ (AOR = 2.734, 95%CI: 1.01–7.395; P<0.048). Likewise, the odds of death from MDR-TB among female patients with MDR-TB was significantly higher than the odds of death among male patients with MDR-TB (AOR = 2.511, 95%CI: 1.005–6.272; P<0.049). In summary, about 26% of the total deaths from MDR-TB, observed in this study, was explained by the presence of some co-morbidity with MDR-TB at the baseline, and being a female patient with MDR-TB (Nagelkerke R Square = 0.257).

**Table 4:** Results of the multivariable analysis using logistic regression on factors associated with unfavourable MDR-TB treatment outcome of the study participants at Adama and Nekemte referral hospitals, Oromia, Ethiopia, December 2012 –September 2016.

Variable	Crude OR^@^	95% CI^@@^	P-value	Adjusted OR	95% CI	P-value
Presence of any co-morbidity at the baseline	2.864	1.217–6.743	0.016	4.260	1.607–11.297	0.004
Body mass index (BMI)	2.925	1.176–7.277	0.021	2.734	1.01–7.395	0.048
Sex	2.436	1.066–5.566	0.035	2.511	1.005–6.272	0.049
HIV	2.7.41	1.112–6.761	0.029	0.088		0.767
Drug-resistance type	2.635	1.088–6.384	0.032	2.630		0.105

^@ = Odds Ratio;^

^@@ = Confidence Interval.^

### Respiratory MDR-TB infection control practices at the study sites

From the total of 114 (n = 114) patients who lived with at least one househld contact, tracing and evaluation of contacts was conducted only for 60 (53%) patients. For the remaining 54 (47%) patients, it was unknown whether any of their household contacts were traced. Assessment of respiratory MDR-TB infection control practices during patients’ movement between the hospitals and community level treatment follow-up centres showed that, during the initial patient linkage to the community 97 (92%) (n = 105) of the patients were transported using hospital ambulances and were also escorted by the nurse caregivers at the hospitals. After initial patient linkage to the community, patients used public transport services during their travels to hospitals to attend the monthly MDR-TB follow-up services. Moreover, patients from the remote rural areas use public transport to attend treatment at the community treatment follow-up centres. Analysis of respiratory MDR-TB infection control practice at the patients’ household level revealed that, for 105 (77%) patients who live with family, caregivers did not visit the patients’ homes to educate patient families on the risk of respiratory MDR-TB transmission and make housing arrangement before patients are sent back home. This study revealed that 8 (6%) of the total patients included in the study were diagnosed among household contacts of index patients with MDR-TB.

## Discussions

At six months after commencing the treatment for MDR-TB, 72% of the patients were culture negative, while 19% died of the disease. The 72% culture negative rate by the end of six months is more than the 62% rate of culture conversion reported by Molla et al. among patients treated at treatment centres in the Amhara and Oromia regions of Ethiopia, but the 19% death rate is more than the 10% death rate reported by the same authors [38]. A substantial percentage (31%) of the patients had co-morbidity with MDR-TB, 83% of which was due to HIV. A study conducted in South Africa cited that, compared with HIV-negative patients, HIV-positive patients with MDR-TB had a lower chance of culture conversion and a higher chance of death [49]. In this view, the higher proportion of MDR-TB and HIV co-infection rate among patients included in this study might be a risk factor for the observed higher proportion of death among patients included in this study. It is worth noting that 87% (26/30) of the total deaths revealed in this study occurred during the first six months after commencing treatment, while only 13% of the total deaths occurred during the subsequent 18 months of treatment. This finding signals the need for intensive care during the initial months of patient treatment for MDR-TB.

The 69% composite treatment success rate revealed in this study is less and the 27% death rate is higher respectively than the 75% treatment success rate and 15% death rate reported by Molla et al. [38]. The 69% treatment success rate is also lower than the 78.6% treatments success rate reported by Meressa et al. [39]. However, the result is similar to the 70.6% treatment success rate reported by Anderson et al. [42], According to the report of Anderson et al., HIV co-infection with MDR-TB is associated with a higher rate of death from MDR-TB. In this view, the relatively low treatment success rate compared to the report by Meressa et al. [39], might be due to the higher rate of MDR-TB and HIV/AIDS co-infection observed among patients included in this study. Total of 46 (35%), (n = 132), patients were not employed and 70 (53%) were employed in the informal sector, mainly in daily labour works. This indicates the low socio-economic status of the patients included in this study. It is repeatedly cited in the literature that low monthly household income, living in poverty and unemployment are predictors of poor treatment outcome among patients with MDR-TB [9, 40]. In this view, the high proportion of unemployment among patients included in this study is a potential challenge for patients to adhere to the standard schedule of the treatment given for MDR-TB. Most patients included in this study experienced at least one episode of adverse drug reactions in the course of their treatment. According to the reports by the World Health Organisation (WHO) [41] the presence of some co-morbidity with MDR-TB increases the risk of occurrence of adverse drug reactions from second-line drugs. In the United Kingdom [42] and Nigeria [43], it was documented that the presence of any co-morbidity with MDR-TB is associated with poor treatment outcome and high mortality among patients treated for MDR-TB. Caminero (2013) also cited that for the poor patients with MDR-TB, malnutrition impairs recovery [30]. Another report by the WHO [44] indicated that low body mass index (BMI) and lack of adequate weight gain during treatment are associated with death and increases the chance of occurrence of adverse drug reactions. Similarly, the report by Yuan et al. [45]; Vishakha et al. [46] and that of Lange et al. [47] each indicated that malnutrition is a risk factor for low cure rate and high rate of death among the poor patients with MDR-TB. In this study, the odds of death was significantly higher among patients with BMI less than 18.5kg/m^2^, (AOR = 2.734, 95%CI: 1.01–7.395; P<0.048) and those with some co-morbidity at baseline (AOR = 4.260, 95%CI: 1.607–11.29; p<0.004). In view of extant literature, the high level of co-morbidity and malnutrition associated with MDR-TB revealed in this study could be a risk factor for the observed high proportion of death and high prevalence of adverse drug reactions among patients included in this study.

The result of this study indicates that addressing co-morbidities and MDR-TB associated malnutrition is key to improving treatment outcomes of patients treated for MDR-TB. The absence of optimum respiratory MDR-TB infection control practice at patients’ household level and the use of public transport services by patients, revealed in this study, are potential risk factors for disease transmission among the community. Moreover, community level respiratory MDR-TB infection control effort did not meet the respiratory MDR-TB infection control recommendations of the Federal Ministry of Health of Ethiopia [16]. The observed 6% MDR-TB cases diagnosed among close contacts is more than the 3% to 5.4% reported from Peru [48]. According to the report of Scardigli et al. [49], poor respiratory MDR-TB infection control increases the risk of MDR-TB transmission to close contacts. In high HIV-prevalent settings, the situation amplifies disease occurrence within families. Thus, the poor MDR-TB infection control and the high prevalence of HIV among patients with MDR-TB, revealed in this study, are potential risk factors for an increase in the number of patients with MDR-TB in the community. The 6% prevalence of MDR-TB among close contacts is a warning sign regarding community and household level respiratory MDR-TB infection control in the study areas.

## Conclusion

If the problem of MDR-TB and the factors determining the treatment outcomes of patients with MDR-TB are to be tackled successfully, the factors determining the treatment outcomes of patients with MDR-TB need to be identified. In this regard, this study has identified socio-demographic and clinical factors that determine the treatment outcomes of patients with MDR-TB. Thus, the results from this study will enable health decision makers and caregivers for MDR-TB in Ethiopia to make evidence-informed decisions regarding the MDR-TB programme design and its management and resource allocation decisions during the subsequent national effort to expand the programmatic management of MDR-TB in the country.

## Recommendations

Improving socio-economic and nutritional support and provision of integrated care on MDR-TB and HIV/AIDS is recommended to mitigate the higher level of death among patients treated for MDR-TB. To mitigate the inadvertent transmission of MDR-TB among the community, due emphasis should be given to respiratory MDR-TB infection control efforts. Lastly, the authors recommend further study to investigate factors behind the gender-based differentials of MDR-TB treatment outcomes revealed in this study.

## Supporting information

S1 File (DOCX)

S1 Data (SAV)

## References

[1] Heemskerk D, Caws M, Marais B & Farrar J. Tuberculosis in adults and children. New York: Springer; 2015. 26937536

[2] Zwerling A, Hanrahan C & Dowdy DW. Ancient disease, modern epidemiology: a century of progress in understanding and fighting tuberculosis. Am J Epidemiol. 2016; 183 (5):407–414. doi: 10.1093/aje/kwv176 26865266

[3] Davies PDO, Gordon SB & Davies G, editors. Clinical tuberculosis. 5th edition. London: CRC Press; 2014.

[4] Ribon W, editor. Tuberculosis: expanding knowledge. Published by AvE4EvA. E-ISBN-13:978–953-51-2139-8; 2015.

[5] Munsab A, Santanu M, Revinder M, Pradeep K & Ankur G. Effect of social factors on tuberculosis patients: a comprehensive illness behavior study. IJRAP. 2013; 3(1):123–126.

[6] Lewis JM & Sloan DJ. The role of delamanid in the treatment of drug-resistant tuberculosis. Therapeutics and Clinical Risk Management. 2015; 11:779–791. doi: 10.2147/TCRM.S71076 25999726 PMC4437614

[7] Hatfull GF & Jacobs WR Jr, editors. Molecular genetics of mycobacteria, 2nd edition. Washington, DC: American Society for Microbiology; 2014.

[8] WHO. Global tuberculosis report 2014. Geneva: World Health Organization; 2014.

[9] Dheda K & Migliori GB. The global rise of extensively drug-resistant tuberculosis: is the time to bring back sanatoria now overdue?. Lancet, 2012; 379:773–75. doi: 10.1016/S0140-6736(11)61062-3 22033020

[10] Udwadia ZF. Totally drug resistant-tuberculosis in India: the bad just got worse, J Assoc Chest Physicians. 2016; 4:41–2.

[11] Behera D. Totally drug-resistant tuberculosis—a fact or myth?. Indian J Tuberc. 2012; 59:190–193. 23342538

[12] Dheda K, Gumbo T, Gandhi NR, Murray M, Theron G, Udwadia Z et al. Global control of tuberculosis: from extensively drug-resistant to untreatable tuberculosis. Lancet Respir Med. 2014; (4):321–38. doi: 10.1016/S2213-2600(14)70031-1 24717628 PMC5526327

[13] Tadolini M, Centis R, D’Ambrosio L & Migliori GB. TB and MDR-TB: what is new in 2012?, Breathe. 2012; 9(2):101–111.

[14] WHO. Global tuberculosis report 2016: Geneva: World Health Organization; 2016.

[15] Migliori GB & Sotgiu G. Measuring the effect of tuberculosis control: a step forward. Thelancet., 2014; 383:2026–28. doi: 10.1016/S0140-6736(14)60032-5 24650956

[16] Federal Ministry of Health of Ethiopia. Guidelines on programmatic management of drug resistant tuberculosis in Ethiopia. 2nd edition. Addis Ababa, Ethiopia; 2014.

[17] Falzon D, Jaramillo E, Wares F, Zignol M, Floyd K & Raviglione MC. Universal access to care for multidrug-resistant tuberculosis: an analysis of surveillance data. Lancet Infect Dis. 2013; 13:690–97. doi: 10.1016/S1473-3099(13)70130-0 23743044

[18] WHO. Multidrug and extensively drug-resistant TB (M/XDR-TB): 2010 global report on surveillance and response. Geneva: World Health Organization; 2010.

[19] Kendall EA, Azman AS, Cobelens FG & Dowdy DW. MDR-TB treatment as prevention: The projected population-level impact of expanded treatment for multidrug-resistant tuberculosis, PLoS ONE. 2017; 12(3): e0172748. doi: 10.1371/journal.pone.0172748 28273116 PMC5342197

[20] Biadglegne F, Sack U & Rodloff AC. Multidrug-resistant tuberculosis in Ethiopia: efforts to expand diagnostic services, treatment and care. Antimicrobial Resistance and Infection Control. 2014; 3(31):1–10. doi: 10.1186/2047-2994-3-31 25685333 PMC4328048

[21] Rusen ID, Squire SB & Billo NE. Poverty and lung health. Expert Rev Resp Med. 2010; 4(2):163–165. doi: 10.1586/ers.10.11 20406081

[22] WHO. Guideline: nutritional care and support for patients with tuberculosis. Geneva: World Health Organization; 2013. 24624480

[23] Uplekar M, Weil D, Lonnroth K, Jaramillo E, Lienhardt C, Dias HM et al. WHO’s new End TB Strategy. Lancet. 2015; 385:1799–801. doi: 10.1016/S0140-6736(15)60570-0 25814376

[24] Hichey AJ, editor. Drug delivery systems for tuberculosis prevention and treatment. United Kingdom: John Wiley & Sons; 2016.

[25] Raviglione MC, editor. Tuberculosis fourth edition: the essentials.2010. New York: Informa Healthcare; 2010.

[26] Raviglione MC, Marais B, Floyd K, Lönnroth K, Getahun H, Migliori GB et al. Scaling up interventions to achieve global tuberculosis control: progress and new developments. Lancet. 2012; 379:1902–13. doi: 10.1016/S0140-6736(12)60727-2 22608339

[27] Kurbatova EV, Dalton T, Ershova J, Tupasi T, Caoili JC, Van Der Walt M et al. Additional Drug Resistance of Multidrug-Resistant Tuberculosis in Patients in 9 Countries. Emerging Infectious Diseases. 2015; 21(6):977–983. doi: 10.3201/eid2106.141329 25988299 PMC4451908

[28] Ayles M & Godfrey-Faussett P. Tuberculosis and the human immunodeficiency virus in the International Journal of Tuberculosis and Lung Disease in 2008. Int J Tuberc Lung Dis. 2009; 13(12):1450–1455. 19919761

[29] Gandhi NR, Andrews JR, Brust JCM, Montreuil R, Weissman D, Heo M, et al. Risk factors for mortality among MDR- and XDR-TB patients in a high HIV prevalence setting. INT J TUBERC LUNG DIS. 2012; 16(1):90–97. doi: 10.5588/ijtld.11.0153 22236852 PMC3302205

[30] Caminero JA, editor. Guidelines for clinical and operational management of drug-resistant tuberculosis. Paris, France: International Union Against Tuberculosis and Lung Disease; 2013.

[31] WHO. Guidelines for the programmatic management of drug-resistant tuberculosis: 2011 Update. Geneva: World Health Organization; 2011. 23844450

[32] Van Deun A, Maug AKJ, Salim MAH, Das PK, Sarker MR, Daru P et al. Short, highly effective, and inexpensive standardized treatment of multidrug-resistant tuberculosis. Am J Respir Crit Care Med. 2010; 182:684–692. doi: 10.1164/rccm.201001-0077OC 20442432

[33] Siroka K, Ponce NA & Lönnroth K. Association between spending on social protection and tuberculosis burden: a global analysis. Lancet Infect Dis. 2015; 16: 473–79. doi: 10.1016/S1473-3099(15)00401-6 26725446 PMC4834192

[34] Longest BB. Health program management: form development through evaluation. 2^nd^ edition. San Francisco: Jossey-Bass; 2015.

[35] WHO. Companion handbook to the WHO guidelines for the programmatic management of drug- resistant tuberculosis. Geneva: World Health Organization; 2014b. 25320836

[36] CIOMS. International ethical guidelines for health-related research involving humans. Geneva: World Health Organization; 2016. 10.1001/jama.2016.18977 27923072

[37] Singh YK. Fundamentals of research methodology and statistics. New Delhi: New Age International Publishers; 2006.

[38] Molla Y, Jerene D, Jemal I, Nigussie G, Kebede T, Kassie Y et al. The experience of scaling up a decentralized, ambulatory model of care for management of multidrug-resistant tuberculosis in two regions of Ethiopia. Journal of Clinical Tuberculosis and Other Mycobacterial Diseases. 2017; 7:28–33. doi: 10.1016/j.jctube.2017.03.001 31723698 PMC6850264

[39] Meressa D, Hurtado RM, Andrews JR, Diro E, Abato K, Daniel T et al. Achieving high treatment success for multidrug-resistant TB in Africa: initiation and scale-up of MDR-TB care in Ethiopia-an observational cohort study. Thorax. 2015; 70:1181–1188. doi: 10.1136/thoraxjnl-2015-207374 26506854 PMC4680185

[40] Djibuti M, Mirvelashvili E, Makharashvili N & Magee MJ. Household income and poor treatment outcome among patients with tuberculosis in Georgia: a cohort study. BMC Public Health. 2014; 14(88):1–7. doi: 10.1186/1471-2458-14-88 24476154 PMC3908381

[41] WHO. Companion handbook to the WHO guidelines for the programmatic management of drug-resistant tuberculosis. Geneva: World Health Organization; 2014. 25320836

[42] Anderson LF, Tamne S, Brown T, Watson JP, Mullarkey C, Zenner D et al. Transmission of multidrug-resistant tuberculosis in the UK: a cross-sectional molecular and epidemiological study of clustering and contact tracing. Lancet Infect Dis. 2014; 14:406–15. doi: 10.1016/S1473-3099(14)70022-2 24602842

[43] Babatunde OA, Elegbede OE, Ayodele M, Fadare JO, Isinjaye AO, Ibirongbe DO et al. Factors affecting treatment outcomes of tuberculosis in a tertiary health center in Southwestern Nigeria. International Review of Social Sciences and Humanities. 2013; 4(2):209–218.

[44] WHO. Guideline: nutritional care and support for patients with tuberculosis. Geneva: World Health Organization; 2013. 24624480

[45] Yuan X, Zhang T, Kawakami K, Zhu J, Zheng W, Li H et al. Genotyping and clinical characteristics of multidrug and extensively drug-resistant tuberculosis in a tertiary care tuberculosis hospital in China. BMC Infectious. Diseases. 2013; 13(315):1–9. 23849244 10.1186/1471-2334-13-315 PMC3716566

[46] Vishakha KK & Sanjay BT. Analysis of 63 patients of MDR TB on DOTS plus regimen: an LG hospital, TB unit, Ahmedabad experience. GUJARAT MEDICAL JOURNAL. 2013; 68(2):52–57.

[47] Lange C, Abubakar I, Alffenaar JWC, Bothamley G, Caminero JA, Carvalho ACC et al. Management of patients with multidrug-resistant/extensively drug-resistant tuberculosis in Europe: a TBNET consensus statement. Eur Respir J.2014; 44:23–63. doi: 10.1183/09031936.00188313 24659544 PMC4076529

[48] Becerra MC, Appleton SC, Franke MF, Chalco K, Arteaga F & Bayona J 2011. Tuberculosis burden in households of patients with multidrug-resistant and extensively drug-resistant tuberculosis: a retrospective cohort study. Thelancet, 377:147–52. doi: 10.1016/S0140-6736(10)61972-1 21145581 PMC13508782

[49] Scardigli A & Caminero JA. Management of drug-resistant tuberculosis. Curr Respir Care Rep., 2013. 2:208–217.

