# Supplementary material for: Treatment outcomes of patients with MDR-TB and its determinants at referral hospitals in Ethiopia
Source: PLoS One. 2022 Feb 17;17(2):e0262318. doi: 10.1371/journal.pone.0262318 (PMC8853509; doi:10.1371/journal.pone.0262318)
Supplement: S1 File — (DOCX) [file pone.0262318.s001.docx]

# Annexure: Data collection tools: the structured questionnaire

**Part I: Structured questionnaire for the collection of the data on the clinical and programmatic management of patients with MDR-TB**

**General instruction:** Data collector captures data available on MDR-TB patient chart; unit MDR-TB register; patient treatment card. When there is no data filled into any of the sources mentioned for any particular question, write ‘no data’.

| Date Questionnaire filled in: DD/MM/YY: ____________: ___________ |
| --- |
| ______________________________ data collector: _____ |
| First Ever MDR-TB patient registered on facility Register? DD/MM/YY _____________________________________ Last MDR-TB patient registered on facility Register: DD/MM/YY__________________________________________ |

| **Questions to assess programmatic management of drug-resistant tuberculosis at the two study sites**  **Source of data:** Unit MDR-TB register, individual MDR-TB patient chart & MDR-TB patient treatment card. | | | | | | | | | | | | | | | | | | |
| --- | --- | --- | --- | --- | --- | --- | --- | --- | --- | --- | --- | --- | --- | --- | --- | --- | --- | --- |
| **Questions Related to MDR-TB Patient’s Socio-demographic Data** | | | | | | | | | | | | | | | | | | |
| 1. Patient Medical Registration Number (MRN):_______________________ | | | | | | | | | | | | | | | | | | |
| 1. Patient’s unique MDR-TB Registration Number: _____________________ | | | | | | | | | | | | | | | | | | |
| 1. Sex of the Patient: 1. Male 2. Female | | | | | | | | | | | | | | | | | | |
| 1. Age of the patient in completed years____________ | | | | | | | | | | | | | | | | | | |
|  | | | | | | | | | | | | | | | | | | |
| 1. Patient’s employment status. 1. Formally employed 2. Self-employed 3. Unemployed   4. Other (Specify)_____________________ | | | | | | | | | | | | | | | | | | |
| 1. Initial (pre-treatment) Weight (in Kgs): _________________________________ | | | | | | | | | | | | | | | | | | |
| 1. Patient Height in centimetre (CMs) _____________ BMI (kg/m^2^):__________________ | | | | | | | | | | | | | | | | | | |
| 1. Date patient escorted to the MDR-TB Treatment Initiating Centre: _________/ _________/_________ (Date/Month/Year) | | | | | | | | | | | | | | | | | | |
| 1. Date patient initiated on second-line drugs: ____/_______/_____(Date/Month/Year) | | | | | | | | | | | | | | | | | | |
| 1. Does the TB patient have designated treatment supporter outside the TIC? 1. Yes 2. No 3. Unknown [if ‘No' skip to 13] | | | | | | | | | | | | | | | | | | |
| 1. If yes to question no. 11, who is the patient’s treatment supporter? 1. Caregiver at TFC 2. Health Extension worker 3. Family member 4. Other (specify)_______________________ | | | | | | | | | | | | | | | | | | |
| **Current MDR-TB related Information of the Patient** | | | | | | | | | | | | | | | | | | |
| 1. What diagnostic method(s) was/were/ used to diagnose the patient with MDR-TB? [circle all that apply] **1**. Bacteriology (Smear microscopy) **2**. Bacteriology (culture) **3.** Genotypic (using GeneXpert) **4**. Genotypic (using Line Probe Assay) 5. Clinical (CXR & histopathology) 7**.** Other (specify)________________________________________ | | | | | | | | | | | | | | | | | | |
| 1. Site of the TB Disease: 1. Pulmonary 2. Extra pulmonary 3. Both pulmonary & Extra pulmonary TB | | | | | | | | | | | | | | | | | | |
| 1. What is the type of the TB case? **1**. Bacteriologically confirmed pulmonary TB **2.** Bacteriologically confirmed extra pulmonary TB **3.** Clinically diagnosed pulmonary TB 4**.** Clinically diagnosed extra pulmonary TB 5. Other (specify)_____________________ | | | | | | | | | | | | | | | | | | |
| 1. If TB is pulmonary and sputum smear examination was done, what is the result of the diagnostic sputum smear examination? 1. Smear Positive 2. Smear Negative 3. Unknown | | | | | | | | | | | | | | | | | | |
| 1. If TB is pulmonary and diagnostic sputum was done, what was the semi-quantitative bacillary load reported at diagnosis? 1. **No AFB** (Negative)=0 AFB /100 HPF 2. **Scanty** (1+) =1-9 AFB/ 100 HPF 3. **Moderate** (2+) =10-99 AFB/100HPF 4. **High** (3+) = (1-10 AFB/1HPF/ 5. **Very High** (4+)/>10 AFB/1 HPF/ | | | | | | | | | | | | | | | | | | |
| **Use of Diagnostic Radiological Examination (Instruction:** Data source is individual patient file/chart) | | | | | | | | | | | | | | | | | | |
| 1. Was diagnostic radiological examination used for the patient? 1. Yes 2. No 3. Unknown [If ‘No’ or ‘Unknown’, skip to question 21] | | | | | | | | | | | | | | | | | | |
| 1. If diagnostic radiography was used, what the extent of the baseline lung disease was as revealed by radiography: 1. Normal 2. Unilateral lesion 3. Bilateral lesion 4. Cavitation 5. Fibrosis 6. Other finding (specify)___________________________________ | | | | | | | | | | | | | | | | | | |
| 1. If there was lung cavitary lesion at baseline, what is the extent of the cavitary lesion? 1. Unilateral 2. Bilateral 3. Other type (specify) __________________ **NB:** This data is collected from individual patient medical file/patient chart/. | | | | | | | | | | | | | | | | | | |
| 1. What is the TB patient’s resistance type: 1. RR 2. MDR-TB 3. Pre-XDR-TB 4. XDR-TB 5. Poly-resistant 6. Unknown | | | | | | | | | | | | | | | | | | |
| 1. What is the MDR-TB patient’s Registration group? 1. New 2. Relapse 3. Treatment after lost to follow ups 4. Treatment after failure of new regimen 5. Treatment after failure of re-treatment 6. Transfer in patient (T) 7. Other previously treated TB (O) | | | | | | | | | | | | | | | | | | |
| 1. Does the patient have history of treatment with regimen containing any of the second-line anti-tuberculosis drugs? 1. Yes 2. No 3. Not known (If ‘No’ skip to Question 25) | | | | | | | | | | | | | | | | | | |
| 1. If the patient has history of previous treatment with regimen containing second-line drugs, what was the patient’s treatment outcome during treatment with regimen containing second-line drugs? 1. Cured 2. Treatment Completed 3. Treatment Failed 4. Lost to Follow Ups 5. Not evaluated (not known) | | | | | | | | | | | | | | | | | | |
| 1. Is there any co-morbid condition at baseline? 1. Yes 2. No 3. Unknown (If ‘No’ skip to question 27) | | | | | | | | | | | | | | | | | | |
| 1. If there is any co-morbidity at baseline, what was the co-morbid condition? 1. Diabetes 2. Kidney Diseases 3. Hypertension 4. COPD 5. Liver Disease 6.HIV/AIDS 7. Psychiatric illness 8.HIV/AIDS related opportunistic infection (OIs) 9. Seizers 10. Other co-morbidities (specify)____________________________________________ | | | | | | | | | | | | | | | | | | |
| 1. Is there any co-morbidity diagnosed in the course of patient treatment for MDR-TB? 1. Yes 2. No 3. Unknown (NB: This co-morbidity may be newly diagnosed for patients without co-morbidity at baseline & additional co-morbidity for patients with any co-morbidity at baseline) | | | | | | | | | | | | | | | | | | |
| 1. If there is any co-morbidity diagnosed in the course of patient treatment, what was the co-morbidity? 1. Diabetes 2. Kidney diseases 3. Hypertension 4. Liver Disease 5. Psychiatric illness 6. Seizers 7.Other(specify) _______________________________ | | | | | | | | | | | | | | | | | | |
| **Questions related to practice of tracing household & close contacts of the index patient with MDR-TB** | | | | | | | | | | | | | | | | | | |
| 1. Number of household/close/ contacts living with the index patient. 1. None (alone) 2. 1-3 persons 3. 4-6 persons 4. 7-8 persons 5. 9-10 persons 6. Not Known (no evidence at TIC) | | | | | | | | | | | | | | | | | | |
| 1. If the patient has contacts, are any of the contacts of the index MDR-TB patient traced? 1. Yes 2. No 3. Unknown (No evidence at the TIC) 4. Other practice (specify)_____________________________(If ‘No’ or ‘Unknown’, skip to question 38 below) | | | | | | | | | | | | | | | | | | |
| 1. If yes to question 30, how many household or close contacts of the index MDR-TB patient were traced? ____________________ | | | | | | | | | | | | | | | | | | |
| 1. If yes to question 30, how many of the traced household or close contacts were evaluated for TB clinically or through lab? _____ | | | | | | | | | | | | | | | | | | |
| 1. If yes to question 30, were there contacts screen positive for TB (presumptive TB)? 1. Yes 2. No [if ‘no’ skip to # 38] | | | | | | | | | | | | | | | | | | |
| 1. Is DST done for contacts those found to be screen positive for TB? 1. Yes 2. No 3. Unknown | | | | | | | | | | | | | | | | | | |
| 1. If DST was done for TB screen positive contacts, answer questions 35.1-35.5 (# of answers determined by # of DST available) | | | | | | | | | | | | | | | | | | |
| - 1. DST result for contact 1? **1**. No MTB **2**. MTB detected but no RR/MDR **3**. RR/MDR detected **4**. Indeterminate result   2. DST result for contact 2? **1**. No MTB **2**. MTB detected but no RR/MDR **3**. RR/MDR detected **4**. Indeterminate result   3. DST result for contact 3? **1**. No MTB **2**. MTB detected but no RR/MDR **3**. RR/MDR detected **4**. Indeterminate result   4. DST result for contact 4? **1**. No MTB **2**. MTB detected but no RR/MDR **3**. RR/MDR detected **4**. Indeterminate result   5. DST result for contact 5? **1**. No MTB **2**. MTB detected but no RR/MDR **3**. RR/MDR detected **4**. Indeterminate result | | | | | | | | | | | | | | | | | | |
| 1. How many of the clinically or lab evaluated contacts of the index RR/MDR-TB patient were diagnosed with susceptible TB _____ | | | | | | | | | | | | | | | | | | |
| 1. How many of the clinically or lab evaluated contacts of index RR/MDR-TB were diagnosed with RR/MDR-TB? _______________ | | | | | | | | | | | | | | | | | | |
| 1. If there is practice of tracing household and close contacts, what is the frequency of evaluation of contacts of known RR/MDR-TB patients? 1.Done only once 2.Quarterly 3.Every six month 4.Every year 5.Other schedule (specify) _____________________ | | | | | | | | | | | | | | | | | | |
| 1. For how long is a household/close/ contact of a confirmed RR/MDR-TB patient is followed? 1. For six months 2. For one year 3. For two years 4. For three years 5. For four years 6. Other practice or schedule (specify)________________________________ | | | | | | | | | | | | | | | | | | |
| 1. Result of drug-susceptibility testing (DST) for the patient: Enter all available DST results for the specified anti-tuberculosis drugs. [**Note:** R=Resistant; S= Susceptible; I= Indeterminate; U= DST result unknown or not done ] | | | | | | | | | | | | | | | | | | |
| Drug | R | H | E | S | KM | Cm | Ofx | Am | Lfx | Mfx | Eto | Pto | Cs | PAS | Other | Other | Other |  |
| Resistance status |  |  |  |  |  |  |  |  |  |  |  |  |  |  |  |  |  |  |

1. Date intensive phase MDR-TB treatment started (DD/MM/YY) __________________
2. What is the MDR-TB regimen that the patient is taking (took) during intensive phase: (write the regimen, that is, drugs and duration) ______________________________
3. What is the number of presumed effective second-line drugs used in the patient’s MDR-TB treatment regimen during intensive phase (**NB**: do not count any first-line anti-tuberculosis drugs included in the regimen as one of presumed effective drug)?
4. 2 drugs 2. 3 drugs **3**. 4 drugs **4.** 5 drugs **5**. Other (specify)________________
5. Total # of daily tablets given to the patient in the second-line regimen during intensive phase (include tablets of ancillary drugs, if any): ____________________________
6. Date continuation phase MDR-TB treatment started (DD/MM/YY) ____________ [If patient died before entering continuation phase, skip to question 49]
7. What is the MDR-TB regimen that the patient is taking (took) during continuation phase: (write regimen that is drugs and duration)_____________________________
8. What is the number of presumed effective second-line drugs used in the patients’ MDR-TB treatment regimen during continuation phase? **1**. 2 drugs **2**. 3 drugs **3**. 4 drugs **4**. 5 drugs **5**. Other (Specify)_____________________________________
9. Total # of daily tablets given to the patient in the SLD regimen during continuation phase (include tablets of ancillary drugs, if any): ____________________________________________________________________
10. MDR-TB patient’s Daily Observed Treatment (DOT) attendance: [**Instruction:** Note that the box is subdivided into upper and lower parts to fill in Daily Observed Treatment status for morning and evening does respectively in case a drug is given in divided doses. If daily dose of a given drug is given once, use upper box. Fill in: 3=if dose taken is Directly Observed by treatment supporter; 2=if dose is taken by patient but not directly observed by treatment supporter and 1= if dose of the day not taken by the patient.

|  | DAYS IN A MONTH (ETHIOPIAN CALENDAR) | | | | | | | | | | | | | | | | | | | | | | | | | | | | | |
| --- | --- | --- | --- | --- | --- | --- | --- | --- | --- | --- | --- | --- | --- | --- | --- | --- | --- | --- | --- | --- | --- | --- | --- | --- | --- | --- | --- | --- | --- | --- |
| Month | 1 | 2 | 3 | 4 | 5 | 6 | 7 | 8 | 9 | 10 | 11 | 12 | 13 | 14 | 15 | 16 | 17 | 18 | 19 | 20 | 21 | 22 | 23 | 24 | 25 | 26 | 27 | 28 | 29 | 30 |
| 0 |  |  |  |  |  |  |  |  |  |  |  |  |  |  |  |  |  |  |  |  |  |  |  |  |  |  |  |  |  |  |
| 1 |  |  |  |  |  |  |  |  |  |  |  |  |  |  |  |  |  |  |  |  |  |  |  |  |  |  |  |  |  |  |
| 2 |  |  |  |  |  |  |  |  |  |  |  |  |  |  |  |  |  |  |  |  |  |  |  |  |  |  |  |  |  |  |
| 3 |  |  |  |  |  |  |  |  |  |  |  |  |  |  |  |  |  |  |  |  |  |  |  |  |  |  |  |  |  |  |
| 4 |  |  |  |  |  |  |  |  |  |  |  |  |  |  |  |  |  |  |  |  |  |  |  |  |  |  |  |  |  |  |
| 5 |  |  |  |  |  |  |  |  |  |  |  |  |  |  |  |  |  |  |  |  |  |  |  |  |  |  |  |  |  |  |
| 6 |  |  |  |  |  |  |  |  |  |  |  |  |  |  |  |  |  |  |  |  |  |  |  |  |  |  |  |  |  |  |
| 7 |  |  |  |  |  |  |  |  |  |  |  |  |  |  |  |  |  |  |  |  |  |  |  |  |  |  |  |  |  |  |
| 8 |  |  |  |  |  |  |  |  |  |  |  |  |  |  |  |  |  |  |  |  |  |  |  |  |  |  |  |  |  |  |
| 9 |  |  |  |  |  |  |  |  |  |  |  |  |  |  |  |  |  |  |  |  |  |  |  |  |  |  |  |  |  |  |
| 10 |  |  |  |  |  |  |  |  |  |  |  |  |  |  |  |  |  |  |  |  |  |  |  |  |  |  |  |  |  |  |
| 11 |  |  |  |  |  |  |  |  |  |  |  |  |  |  |  |  |  |  |  |  |  |  |  |  |  |  |  |  |  |  |
| 12 |  |  |  |  |  |  |  |  |  |  |  |  |  |  |  |  |  |  |  |  |  |  |  |  |  |  |  |  |  |  |
| 13 |  |  |  |  |  |  |  |  |  |  |  |  |  |  |  |  |  |  |  |  |  |  |  |  |  |  |  |  |  |  |
| 14 |  |  |  |  |  |  |  |  |  |  |  |  |  |  |  |  |  |  |  |  |  |  |  |  |  |  |  |  |  |  |
| 15 |  |  |  |  |  |  |  |  |  |  |  |  |  |  |  |  |  |  |  |  |  |  |  |  |  |  |  |  |  |  |
| 16 |  |  |  |  |  |  |  |  |  |  |  |  |  |  |  |  |  |  |  |  |  |  |  |  |  |  |  |  |  |  |
| 17 |  |  |  |  |  |  |  |  |  |  |  |  |  |  |  |  |  |  |  |  |  |  |  |  |  |  |  |  |  |  |
| 18 |  |  |  |  |  |  |  |  |  |  |  |  |  |  |  |  |  |  |  |  |  |  |  |  |  |  |  |  |  |  |
| 19 |  |  |  |  |  |  |  |  |  |  |  |  |  |  |  |  |  |  |  |  |  |  |  |  |  |  |  |  |  |  |
| 20 |  |  |  |  |  |  |  |  |  |  |  |  |  |  |  |  |  |  |  |  |  |  |  |  |  |  |  |  |  |  |
| 21 |  |  |  |  |  |  |  |  |  |  |  |  |  |  |  |  |  |  |  |  |  |  |  |  |  |  |  |  |  |  |
| 22 |  |  |  |  |  |  |  |  |  |  |  |  |  |  |  |  |  |  |  |  |  |  |  |  |  |  |  |  |  |  |
| 23 |  |  |  |  |  |  |  |  |  |  |  |  |  |  |  |  |  |  |  |  |  |  |  |  |  |  |  |  |  |  |
| 24 |  |  |  |  |  |  |  |  |  |  |  |  |  |  |  |  |  |  |  |  |  |  |  |  |  |  |  |  |  |  |

| 1. Has the patient ever missed the daily dose of SLD? 1. Yes 2. No 3. Unknown (If ‘No’ or ‘Unknown’ skip to question 53). |
| --- |
| 1. If yes to question # 50, what was the number of daily doses of SLDs missed? __________ |
| 1. If yes to question # 50, what was the reason for missing the doses? 1. Drug stock out 2. Patient failure to come for appointment 3. Drug-related adverse reactions 4. Other reason (specify)__________________________________________ |
| 1. Did the patient have history of treatment interruption (treatment discontinuation for less than 2 months) while on MDR-TB treatment? 1. Yes 2. No 3. Unknown |
| 1. Did the patient have history of lost to follow ups (treatment discontinuation for two months or more) while on MDR-TB treatment? 1. Yes 2. No 3. Unknown |
| 1. Is the patient tested for HIV? 1. Yes 2. No 3. Unknown (If ‘No’ skip to 63) |
| 1. If tested for HIV, date HIV test done (DD/MM/YY) ________/______/______ |
| 1. If tested for HIV, HIV test Result of the patient. 1. Positive 2. Negative 3. Indeterminate (If answer is ‘2’ skip to question # 63 |
| 1. If patient was positive for HIV, what was the baseline T-lymphocyte cell bearing (CD4) count (cells/mm3): ________________ |
| 1. If patient was positive for HIV, was the patient given cotrimoxazole preventive therapy (CPT)? 1.Yes 2. No 3.Unknown (If ‘No’ skip to # 61) |
| 1. If cotrimoxazole preventive therapy was given, Date the cotrimoxazole preventive therapy was started (DD/MM/YY) ________/______/______ |
| 1. If Positive for HIV was patient initiated on ART? 1. Yes 2. No 3. Unknown (If ‘No’ skip to # 63) |
| 1. If ART was initiated, Date ART started (DD/MM/YY) ________/______/______ |

**Questions related to assessing MDR-TB Patients’ Bacteriological & Radiological follow up service status.**

1. **MDR-TB patient’s Bacteriologic (sputum smear and culture) follow up status and its result:**

**Instruction:** Write ‘N’ for Culture (C) Negative result; ‘P’ for Culture (C) Positive Result; ‘N’ for sputum (S) negative result and ‘P’ for sputum (S) positive result and ‘ND’ if test not done or result not available both for culture and sputum for a scheduled month. **NB:** Date Specimen collected from a patient for a given follow up month is the same as date of follow up culture & sputum result of that month.

| Type of Follow up | MONTH (0-24 month) | | | | | | | | | | | | | | | | | | | | | | | | |
| --- | --- | --- | --- | --- | --- | --- | --- | --- | --- | --- | --- | --- | --- | --- | --- | --- | --- | --- | --- | --- | --- | --- | --- | --- | --- |
|  | 0 | 1 | 2 | 3 | 4 | 5 | 6 | 7 | 8 | 9 | 10 | 11 | 12 | 13 | 14 | 15 | 16 | 17 | 18 | 19 | 20 | 21 | 22 | 23 | 24 |
| Sputum (S) |  |  |  |  |  |  |  |  |  |  |  |  |  |  |  |  |  |  |  |  |  |  |  |  |  |
| Culture (C) |  |  |  |  |  |  |  |  |  |  |  |  |  |  |  |  |  |  |  |  |  |  |  |  |  |

1. **Patient’s Radiological Follow up status and its result (If 2 or 3 skip to 66):**

| Availability of follow up radiological exam at each phase.  For each column fill: [1=If Done/available/ 2=If not done 3=Unknown (no data)] | | |
| --- | --- | --- |
| At Baseline | At End of Intensive Phase | At End of Treatment |
|  |  |  |

1. Result of Follow up Radiological examination at each scheduled follow up time:

| Result of Follow up Radiological exams at each phase: [Fill in: 1=Improved; 2=No change; 3=Deteriorated; 4=follow up result not available | | |
| --- | --- | --- |
| At Baseline | At End of Intensive Phase | At End of Treatment |
|  |  |  |

1. If there was cavitary lesion at baseline (answer option ‘4’on Q19), what are the subsequent radiological changes in the lung cavitary lesion during scheduled radiological follow-ups? [Fill in: 1=Improved; 2=No change; 3=Deteriorated 4=follow up result not available (If there was no cavitary lesion at baseline skip to 67).

| Level of Lung Cavities at each radiological examination | | |
| --- | --- | --- |
| At Baseline | At End of Intensive Phase | At End of Treatment |
|  |  |  |

**Questions to assess availability of continuum of care for patients with MDR-TB**

1. If the patient was linked to catchment MDR-TB treatment follow up centres (TFCs), ask the following questions and if the patient is treated at TIC, that is ‘No’ to question # 11, skip to question # 68]

| **Note for data collector:** The following activities are expected to be performed for an MDR-TB patient linked to TFCs. Data is obtained from individual patient file and from interview with TIC MDR-TB focal person (nurse): |
| --- |
| - 1. Has contact tracing been completed for the patient? 1. Yes 2. No 3. Unknown (no evidence) |
| - 1. Has the discharge summary been completed for the patient? 1. Yes 2. No 3. Unknown (no evidence) |
| - 1. Are all SLDs related adverse event issues addressed for the patient linked to TFC? 1. Yes 2. No 3. Unknown (If treated at Tic, skip to 67.23). |
| - 1. Have housing arrangements been confirmed for the patient? 1. Yes 2. No 3. Not known (no evidence) |
| - 1. Have household level TB infection control arrangements been confirmed for the patient? 1. Yes 2. No 3. Unknown |
| - 1. How is the patient taken to TFC? 1. Escorted by TIC level caregivers; 2. Escorted by TFC level caregivers; 3.Escorted by immediate public health office; 4.Patient sent alone 5. Other means (specify) ____________________________________ |
| - 1. Has a copy of the patient’s treatment record been handed over to the patient or future care giver? 1. Yes 2. No 3. Unknown |
| - 1. On date of discharge has the date of the first follow-up appointment been arranged for the patient. 1. Yes 2. No 3. Unknown |
| - 1. Is the list of current medication (drugs) known to the patient? 1. Yes 2. No 3. Unknown (no evidence) |
| - 1. Is the list of current medication (drugs) known to the caregiver at TFC? 1. Yes 2. No 3. Unknown (no evidence) |
| - 1. For patients linked to TFCs, has access to medication been secured? 1. Yes 2. No 3. Unknown (no evidence) |
| - 1. Has the Daily Observed Treatment support been organized for the patient at TFC? 1. Yes 2. No 3. Unknown |
| - 1. Level where this patient gets Daily Observed Treatment support? 1. Health centre 2. Community/health post/ 3. Other (specify) ____________________________ |
| - 1. Is there confidence/evidence of certainty/ that the patient will continue taking the medication? 1. Yes 2. No 3. Unknown |
| - 1. Has a hospital contact number/person/ been handed over to the patient for advice? 1. Yes 2. No 3. Unknown |
| - 1. Has a hospital contact number/person/ been handed over to caregiver at the TFC for advice? 1. Yes 2. No 3. Unknown |
| - 1. Is the contact detail of the TB treatment supporter at TFC known to the hospital care giver? 1. Yes 2. No |
| - 1. Is the patient’s contact address known to the hospital care giver? 1. Yes 2. No 3. Unknown |
| - 1. Is the patient’s address known to the immediate public health office? 1. Yes 2. No 3. Unknown |
| - 1. Are treatment support services (e.g. nutrition & house rent, transport) available for the patient? 1. Yes 2. No 3. Unknown |
| - 1. Is the patient aware of the monitoring schedule during the outpatient phase of treatment? 1. Yes 2. No 3. Unknown |
| - 1. Is the care giver at TFC aware of the monitoring schedule during the outpatient phase of treatment? 1. Yes 2. No 3. Unknown |
| - 1. Do the hospital MDR-TB physician(s) have supportive contact with the national MDR/XDR-TB consilium (consulting group of professionals)? 1. Yes 2. No 3. Not known |
| - 1. If ‘yes’ to 67.23, what are the purpose of contact? 1. Management of difficult cases 2. Treatment of adverse drug reactions 3. Drugs and supplies related 4. Other (specify)___________________________________________________________ |

**Questions to assess adverse events associated with treatment with second-line anti-tuberculosis drugs.**

1. Adverse drug associated with second-line drugs [**source of data**: MDR-TB Patient Treatment Card & Individual Patient Chart/file]

| **Instruction: ‘1’ in** the appropriate cell when the specified adverse drug reaction occurs & ‘2’ when the adverse drug reaction does not occur at specified month | **Months of MDR-TB treatment** | | | | | | | | | | | | | | | | | | | | | | | | |
| --- | --- | --- | --- | --- | --- | --- | --- | --- | --- | --- | --- | --- | --- | --- | --- | --- | --- | --- | --- | --- | --- | --- | --- | --- | --- |
| **Months of treatment** | 0 | 1 | 2 | 3 | 4 | 5 | 6 | 7 | 8 | 9 | 10 | 11 | 12 | 13 | 14 | 15 | 16 | 17 | 18 | 19 | 20 | 21 | 22 | 23 | 24 |
| 1. **Gastrointestinal Disorders** | | | | | | | | | | | | | | | | | | | | | | | | | |
| Nausea & Vomiting |  |  |  |  |  |  |  |  |  |  |  |  |  |  |  |  |  |  |  |  |  |  |  |  |  |
| Abdominal pain |  |  |  |  |  |  |  |  |  |  |  |  |  |  |  |  |  |  |  |  |  |  |  |  |  |
| Diarrhea |  |  |  |  |  |  |  |  |  |  |  |  |  |  |  |  |  |  |  |  |  |  |  |  |  |
| Anorexia/appetite loss |  |  |  |  |  |  |  |  |  |  |  |  |  |  |  |  |  |  |  |  |  |  |  |  |  |
| Gastritis |  |  |  |  |  |  |  |  |  |  |  |  |  |  |  |  |  |  |  |  |  |  |  |  |  |
| Pubtic ulcer disease |  |  |  |  |  |  |  |  |  |  |  |  |  |  |  |  |  |  |  |  |  |  |  |  |  |
| 1. **Vestibular/ Ear / Disorders** | | | | | | | | | | | | | | | | | | | | | | | | | |
| Dizziness |  |  |  |  |  |  |  |  |  |  |  |  |  |  |  |  |  |  |  |  |  |  |  |  |  |
| Problem of imbalance |  |  |  |  |  |  |  |  |  |  |  |  |  |  |  |  |  |  |  |  |  |  |  |  |  |
| Hearing loss |  |  |  |  |  |  |  |  |  |  |  |  |  |  |  |  |  |  |  |  |  |  |  |  |  |
| 1. **Eye Related Disorders** | | | | | | | | | | | | | | | | | | | | | | | | | |
| Blurred vision |  |  |  |  |  |  |  |  |  |  |  |  |  |  |  |  |  |  |  |  |  |  |  |  |  |
| Photophobia |  |  |  |  |  |  |  |  |  |  |  |  |  |  |  |  |  |  |  |  |  |  |  |  |  |
| Decreased visual acuity |  |  |  |  |  |  |  |  |  |  |  |  |  |  |  |  |  |  |  |  |  |  |  |  |  |
| 1. **Changes in clinical chemistry** | | | | | | | | | | | | | | | | | | | | | | | | | |
| Decreased K, Ca |  |  |  |  |  |  |  |  |  |  |  |  |  |  |  |  |  |  |  |  |  |  |  |  |  |
| Elevated ALT |  |  |  |  |  |  |  |  |  |  |  |  |  |  |  |  |  |  |  |  |  |  |  |  |  |
| Elevated creatinine |  |  |  |  |  |  |  |  |  |  |  |  |  |  |  |  |  |  |  |  |  |  |  |  |  |
| Elevated uric acid |  |  |  |  |  |  |  |  |  |  |  |  |  |  |  |  |  |  |  |  |  |  |  |  |  |
| hypomagnesemia |  |  |  |  |  |  |  |  |  |  |  |  |  |  |  |  |  |  |  |  |  |  |  |  |  |
| Hypothyroidism (TSH) |  |  |  |  |  |  |  |  |  |  |  |  |  |  |  |  |  |  |  |  |  |  |  |  |  |
| 1. **Musculo-skeletal disorders** | | | | | | | | | | | | | | | | | | | | | | | | | |
| Myalgia (muscle pain) |  |  |  |  |  |  |  |  |  |  |  |  |  |  |  |  |  |  |  |  |  |  |  |  |  |
| Arthralgia (joint pain) |  |  |  |  |  |  |  |  |  |  |  |  |  |  |  |  |  |  |  |  |  |  |  |  |  |
| Arthritis (inflammation involving the joint) |  |  |  |  |  |  |  |  |  |  |  |  |  |  |  |  |  |  |  |  |  |  |  |  |  |
| 1. **Neurological Disorders** | | | | | | | | | | | | | | | | | | | | | | | | | |
| Dysgeusia (Metallic taste ) |  |  |  |  |  |  |  |  |  |  |  |  |  |  |  |  |  |  |  |  |  |  |  |  |  |
| Peripheral neuropathy |  |  |  |  |  |  |  |  |  |  |  |  |  |  |  |  |  |  |  |  |  |  |  |  |  |
| Headache |  |  |  |  |  |  |  |  |  |  |  |  |  |  |  |  |  |  |  |  |  |  |  |  |  |
| Seizures |  |  |  |  |  |  |  |  |  |  |  |  |  |  |  |  |  |  |  |  |  |  |  |  |  |
| 1. **Psychiatric Disorders** | | | | | | | | | | | | | | | | | | | | | | | | | |
| Anxiety |  |  |  |  |  |  |  |  |  |  |  |  |  |  |  |  |  |  |  |  |  |  |  |  |  |
| Insomnia |  |  |  |  |  |  |  |  |  |  |  |  |  |  |  |  |  |  |  |  |  |  |  |  |  |
| Psychosis |  |  |  |  |  |  |  |  |  |  |  |  |  |  |  |  |  |  |  |  |  |  |  |  |  |
| Depression |  |  |  |  |  |  |  |  |  |  |  |  |  |  |  |  |  |  |  |  |  |  |  |  |  |
| Suicidal attempts |  |  |  |  |  |  |  |  |  |  |  |  |  |  |  |  |  |  |  |  |  |  |  |  |  |
| 1. **Dermatological Disorders** | | | | | | | | | | | | | | | | | | | | | | | | | |
| Rash |  |  |  |  |  |  |  |  |  |  |  |  |  |  |  |  |  |  |  |  |  |  |  |  |  |
| Pruritus (itching) |  |  |  |  |  |  |  |  |  |  |  |  |  |  |  |  |  |  |  |  |  |  |  |  |  |
| Pain at site of injection |  |  |  |  |  |  |  |  |  |  |  |  |  |  |  |  |  |  |  |  |  |  |  |  |  |
| 1. **Cardiovascular Related Disorder** | | | | | | | | | | | | | | | | | | | | | | | | | |
| Palpitation |  |  |  |  |  |  |  |  |  |  |  |  |  |  |  |  |  |  |  |  |  |  |  |  |  |
| Generalized weakness |  |  |  |  |  |  |  |  |  |  |  |  |  |  |  |  |  |  |  |  |  |  |  |  |  |
| Cor- pulmonale |  |  |  |  |  |  |  |  |  |  |  |  |  |  |  |  |  |  |  |  |  |  |  |  |  |
| Other cardiovascular disorders |  |  |  |  |  |  |  |  |  |  |  |  |  |  |  |  |  |  |  |  |  |  |  |  |  |
| 1. **Hypersensitivity reactions/immune related** | | | | | | | | | | | | | | | | | | | | | | | | | |
| Bronchospasm |  |  |  |  |  |  |  |  |  |  |  |  |  |  |  |  |  |  |  |  |  |  |  |  |  |
| Generalized urticarial/angioedema |  |  |  |  |  |  |  |  |  |  |  |  |  |  |  |  |  |  |  |  |  |  |  |  |  |
| Breathing difficulty |  |  |  |  |  |  |  |  |  |  |  |  |  |  |  |  |  |  |  |  |  |  |  |  |  |
| Anaphylaxis |  |  |  |  |  |  |  |  |  |  |  |  |  |  |  |  |  |  |  |  |  |  |  |  |  |
| Jaundice |  |  |  |  |  |  |  |  |  |  |  |  |  |  |  |  |  |  |  |  |  |  |  |  |  |
| Hemoptysis |  |  |  |  |  |  |  |  |  |  |  |  |  |  |  |  |  |  |  |  |  |  |  |  |  |
| Herpes zoster |  |  |  |  |  |  |  |  |  |  |  |  |  |  |  |  |  |  |  |  |  |  |  |  |  |
| 1. **Other adverse drug reaction, if any (specify)** | | | | | | | | | | | | | | | | | | | | | | | | | |
|  |  |  |  |  |  |  |  |  |  |  |  |  |  |  |  |  |  |  |  |  |  |  |  |  |  |
|  |  |  |  |  |  |  |  |  |  |  |  |  |  |  |  |  |  |  |  |  |  |  |  |  |  |
|  |  |  |  |  |  |  |  |  |  |  |  |  |  |  |  |  |  |  |  |  |  |  |  |  |  |

1. For this patient has the MDR-TB treatment regimen ever been modified or permanently changed due to adverse drug reaction? 1. Yes 2. No 3. Unknown (If ‘No’ skip to question 71)
2. If the MDR-TB treatment regimen of the patient has ever been modified or permanently changed, what was/were/ the second-line anti-tuberculosis drug suspected? _______________________________________________________________
3. Level of patient access to baseline and follow up clinical laboratory tests [**Instruction** for data collector: The patient weight in Kg is obtained from MDR-TB patient treatment card and the other lab results are attached to individual patient medical record/file so that patient file is source for all other lab test results except for weight; **NB**. At each month lab tests are done for ALT/SGPT, AST/SGOT, Creatinine, K, Ca, TSH, Hgb, WBC and pregnancy test. If test not done write “ND”, if test is done write the actual lab result for that follow up month]

| MONTH | Date | Weight (Kg) | ALT/SGPT | AST/SGOT | Creatinine | Uric Acid | K/Ca | Mg | TSH | Hgb | WBC | Pregnancy Test |
| --- | --- | --- | --- | --- | --- | --- | --- | --- | --- | --- | --- | --- |
| Pre-treatment |  |  |  |  |  |  |  |  |  |  |  |  |
| Month 0 |  |  |  |  |  |  |  |  |  |  |  |  |
| 1 |  |  |  |  |  |  |  |  |  |  |  |  |
| 2 |  |  |  |  |  |  |  |  |  |  |  |  |
| 3 |  |  |  |  |  |  |  |  |  |  |  |  |
| 4 |  |  |  |  |  |  |  |  |  |  |  |  |
| 5 |  |  |  |  |  |  |  |  |  |  |  |  |
| 6 |  |  |  |  |  |  |  |  |  |  |  |  |
| 7 |  |  |  |  |  |  |  |  |  |  |  |  |
| 8 |  |  |  |  |  |  |  |  |  |  |  |  |
| 9 |  |  |  |  |  |  |  |  |  |  |  |  |
| 10 |  |  |  |  |  |  |  |  |  |  |  |  |
| 11 |  |  |  |  |  |  |  |  |  |  |  |  |
| 12 |  |  |  |  |  |  |  |  |  |  |  |  |
| 13 |  |  |  |  |  |  |  |  |  |  |  |  |
| 14 |  |  |  |  |  |  |  |  |  |  |  |  |
| 15 |  |  |  |  |  |  |  |  |  |  |  |  |
| 16 |  |  |  |  |  |  |  |  |  |  |  |  |
| 17 |  |  |  |  |  |  |  |  |  |  |  |  |
| 18 |  |  |  |  |  |  |  |  |  |  |  |  |
| 19 |  |  |  |  |  |  |  |  |  |  |  |  |
| 20 |  |  |  |  |  |  |  |  |  |  |  |  |
| 21 |  |  |  |  |  |  |  |  |  |  |  |  |
| 22 |  |  |  |  |  |  |  |  |  |  |  |  |
| 23 |  |  |  |  |  |  |  |  |  |  |  |  |
| 24 |  |  |  |  |  |  |  |  |  |  |  |  |

1. MDR-TB patient interim treatment outcome at 6 months of follow up? [asked for patients on treatment at least for 6-month] 1. Culture negative 2. Culture positive 3. Patient lost to follow ups 4. Died by 6-month 5. treatment outcome at 6 months not evaluated [If answer is ‘4’ skip to 78]
2. What is current treatment status of the patient? 1. Currently on treatment 2. Treatment stopped/terminated 3. Other (specify)___________________ [If answer is ‘1’, skip to 78]
3. If treatment was stopped/terminated, date treatment stopped (DD/MM/YY) __________
4. Reason for termination of treatment: 1. Treatment successfully completed 2. Died 3. Lost to follow ups 4. Treatment Failed 5. Could not tolerate the regimen (ADRs) 6. Other reason (specify)_________________________________________________________
5. What is the patient’s treatment outcome (ask for those that completed treatment or those for whom treatment outcome is assigned & circle the appropriate answer)? 1. Cured 2. Treatment Completed 3. Treatment Failed 4. Died 5. Lost to Follow Ups 6. Treatment outcome not evaluated.
6. Date MDR-TB treatment outcome assigned for the patient (DD/MM/YY)_____________

***Post treatment follow up services for patients with MDR-TB (facility)***

1. Is there practice of patient follow ups for patients released from treatment after completion of treatment? 1. Yes 2. No (If ‘No’ skip to question 81)
2. If yes to question 78, what is the **frequency** of **follow** **ups**? 1. Monthly 2. Quarterly 3. Bi-annually 4. Annually 5. Other (specify)____________________________________________
3. If yes to question 78, **for how long** are patients followed released from treatment? 1. Only once 2. For one year 3. For two years 4. Other (specify)_____________________________________________________________________
4. Are there cases of relapse among patients released from treatment after completion of treatment? 1. Yes 2. No 3. Unknown
5. Level of completeness and quality of data on each data source (that is Unit MDR-TB register, patient treatment cards, medical files, etc: **1**. Good **2**. Satisfactory **3**. Unsatisfactory **4**. Other observations (specify) ____________________

**Part II: Checklist to assess status of hospitals respiratory TB infection control implementation.**

**[Instruction: General questions on MDR-TB infection control are filled through interview with hospital level focal person for MDR-TB]**

1. Is there a functional l infection prevention (IP) committee in the hospital? 1. Yes 2. No
2. Is the MDR-TB focal person/nurse/ member of the hospital IP Committee? 1. Yes 2. No
3. Is TB infection risk assessment of the facility done and documented for the current fiscal year? 1. Yes 2. No
4. Does the facility have TB infection control plan for the current fiscal year? 1. Yes 2. No
5. Are the health care professionals providing care in the MDR-TB unit trained on TB IP? 1. Yes 2. No
6. Are the non-health care professionals providing care in the MDR-TB unit trained on TB IP? 1. Yes 2. No
7. Is the facility TB IC activity monitored (plan vs performance) and documented? 1. Yes 2. No

**[Instruction: For the following questions, data is filled through observation of hospital’s MDR-TB treatment unit & tape metre is used to measure distance between adjacent beds]**

1. Is there room for isolation of inpatient MDR-TB patients? 1. Yes 2. No
2. If yes to question # 8, what type of MDR-TB patients are isolated? 1. Sputum positives 2. Culture positives 3. All pulmonary MDR-TB cases 4. All type of RR/MDR-TB patients are isolated from one another.
3. If yes to question # 8, is one cohort of inpatient MDR-TB patients isolated from another cohort of MDR-TB patient? 1. Yes 2. No 3. Unknown
4. If no to question # 10, what is the major reason for not practicing isolation of MDR-TB patients of differing cohorts? 1. Absence of adequate room 2. Not usually enforced by the PMDT programme 3. Did not see the danger of infection 4. Other (specify)______
5. Does the inpatient MDR-TB room have adequate cross ventilation (opposite windows/doors open all day)? 1. Yes 2. No
6. Do(es) the inpatient MDR-TB room(s) have access to natural light? 1. Yes 2. No
7. What is the distance between two adjacent beds of two MDR-TB patients? (measure distance from this patient’s bed to all other adjacent beds & record average distance in metres): _________________________________________________________
8. Does each individual inpatient MDR-TB patient have sputum disposal container with proper lid 1. Yes 2. No 3. Unknown
9. Does every MDR-TB patient with pulmonary TB have a face mask? 1. Yes 2. No
10. Is there a shortage of supplies for MDR-TB infection control (N95 & facemasks)? **NB**: according to national guidelines one caregiver that is, nurse/doctor/paramedics needs 2 pieces of N95 per capita per week/? 1. Yes 2. No 3. 4. Unknown
11. What are practical challenges on TB IC in the facility? _____________________
