# Supplementary material for: Treatment outcomes of patients with MDR-TB and its determinants at referral hospitals in Ethiopia
Source: PLoS One. 2022 Feb 17;17(2):e0262318. doi: 10.1371/journal.pone.0262318 (PMC8853509; doi:10.1371/journal.pone.0262318)
Supplement: Attachment — Submitted filename: Review PONE-D-20-39516.pdf [file pone.0262318.s003.pdf]

Review of PONE-D-20-39516

1. The study is based on data from 2012 till September 2016. It is a relatively old data. To make the study more relevant in the present scenario, the authors are advised to include data at least till December 2020.
2. There is discrepancy in data throughout the text, e.g., at one place it is mentioned there are total 30 deaths, at another place it is mentioned it includes 19 males and 15 females that amounts to 34. In table also it is mentioned as 19 and 15. The article should be read thoroughly and the statistical errors need to be resolved.
3. The 'Structural Questionnaire' that was used to collect data should have been shared as an appendix/supplement.
4. The study design is not clear. In one sentence it is written-'both deductive and inductive approaches were used'. However, in the next sentence it is written- 'Only deductive approach was used'. This needs clarification.
5. There is non-uniformity in writing numerical values-at some place in text, it is written as digits and at some places as words.
6. What drug regimen was provided to MDR-TB patients? Is it the same regimen prescribed to all patients or it is different for pulmonary and extrapulmonary cases and also in the same group? If different regimes are given to different patients, then it would be a major confounding factor in analyzing the disease outcome.
7. Does the patient need to pay for the treatment of TB or the drugs are provided free of cost by government of Ethiopia?
8. The reference for classification of initial bacillary load as 'scanty, moderate or high' is required. It needs clear objective definition.
9. Which drug was responsible for suicide due to clinical psychiatric problem as an adverse drug reaction? How the causal relationship was established?
10. The analysis should also be done comparing urban and rural population and its correlation with disease outcome.
11. The English language of the article needs to be corrected and improved.
12. There was no new information in the conclusion of the study. These are already established facts that malnutrition, poor socio-economic status, unemployment, co-infection with HIV and co-morbidities are responsible for poor disease outcome in MDR-TB cases.
