# Supplementary material for: Treatment outcomes of patients with MDR-TB and its determinants at referral hospitals in Ethiopia
Source: PLoS One. 2022 Feb 17;17(2):e0262318. doi: 10.1371/journal.pone.0262318 (PMC8853509; doi:10.1371/journal.pone.0262318)
Supplement: Attachment — Submitted filename: Response to Reviewers.doc.docx [file pone.0262318.s004.docx]

June 22, 2021

Mengistu Kenea Wakjira

Mobile: 251-91-1-30-25-68

P.O.Box: 28,398/1000

E-mail: mengistukenea@gmail.com

Addis Ababa, Ethiopia

The PLOS ONE academic editors/reviewers’ team

Dear Madam/Sr:

Warm Greetings!

This letter is in reference to reviewers’ request to include a rebuttal letter that responds to each point raised by the academic editors and editors and upload it as a separate file. First, I want to owe my heartfelt thanks and appreciations for the reviewers for their smartness and dedication in seeing the manuscript to the very detail to me revisit the data and make corrections. My responses to each point raised by the editors are indicated in the table that follows.

Best regards

Mengistu Kenea Wakjira

# **Table of responses to pints raised by academic editors/reviewers.**

| **Reviewers’ comments** | **Explanation from the authors** |
| --- | --- |
| The period of study is from 26th Dec, 2012 to 17th Sep,2017. During this period a total of 182 patients with MDR-TB were enrolled to treatment of which 46 patients did not meet the inclusion criteria. The inclusion and exclusion criteria should be specified. | I have included the inclusion and exclusion criteria in the current revised manuscript |
| How was the 24 month follow up assessed for patients for patients enrolled in September 2017. | The 24 month follow up assessed for final treatment outcome was made only for n=110 patients. Thus, for those patients enrolled to treatment in in September 2017, 24-month treatment outcome was not assessed (n=26, were active and on treatment by the time of data collection). Thus, the n=26 was excluded from the determination of the 24-month treatment outcome analysis. |
| Abstract- Background- 3^rd^ line may be modified to 'Thus, this study investigated the determinants responsible for poor treatment outcome in patients with MDR-TB at referral hospitals in Ethiopia. | The area of the manuscript is modified in the way it is recommended by the reviewers |
| Abstract- Results-4th line- Delete 'were' after 27%, also elsewhere also in the manuscript | This grammatical edition was corrected through out the contents of the manuscript |
| Patients’ employment status was assigned for (n=132) and there is a category with “other” clarify | As the study relied on archived patients’ data, recorded data on ‘patients’ employment status’ was available for n=132 patients. There was response option labelled as “other”. There were 9 (7%) patients that fallen in this response category. Patient groups that fell in this category included students and those who were not engaged in any formal/informal income generating activity but depend on family income which is common in the Ethiopian culture of extended family relationship. |
| Why only 58 (43%) patients had DST result for both rifampicin and isoniazid (Was DST determined by GeneXpert? If so why….? | GeneXpert is a molecular Diagnostic tool for MDR-TB. It detects tuberculosis and also drug susceptibility test for Rifampicin. In resource constraint countries like Ethiopia that can not widely use the conventional culture test, GeneXpert is used for the diagnosis of MDR-TB. Rifampicin resistant cases of TB are MDR-TB cases and are treated with second-line drugs. This molecular test (GeneXert) currently in use cannot perform drug susceptibility test for Isoniazid. It was for this reason that only 58 (43%) patients had access to DST by conventional culture method and DST result for both rifampicin and isoniazid |
| Table 3- Please correct whether a total of 30 patients died or 34 died? Similarly, comorbidity has been shown as 41/136 in Table 1 and 34 (favourable+unfavourable outcome). Also, HIV positive patients are 34 in Table 1 and 27 in Table 3. | I have admitted that a mistake was made when I was copying the numbers from SPSS output to word document, and I have now corrected it by going back to the SPSS output. I indeed appreciate the reviewers’ dedication in pinpointing such a mistake intime by seeing into the very details.  Now corrected by going back to the database (attached for your check) that from the total of n=136 patients; by the six month 98 (72%) were culture negative while 26 (19%) of the patients were died of the disease and the interim outcome was not evaluated for 12 (9%) of the patients.  By 24^th^ month after commencing treatment 76 (69%) have successfully completed treatment without evidence of failure while 30 (27% ) were died of the disease. |
| Provide a justification for the sample size used in your study, including any relevant power calculations (if applicable) | The major variable among the clinical characteristics of patients with MDR-TB was the presence of co-morbidity with MDR-TB. There were two groups with respect to this. These groups were patients with co-morbidity (p1) and patients without co-morbidity (p2) with MDR-TB at the baseline. From the literature P1 & P2 for this study were, P1=0.81 and p2= 0.70 (Meressa et al 2015:1183). Then power calculation formula for one sample proportion was used to calculate power for the total sample size used for the study. Accordingly, the calculated power for the sample size included in this study was 0.87. Thus, clinical records of all the 136 participants who meet the inclusion criteria were retrieved and included in the study. |
| If your study included minors under 18, state whether you obtained consent form parents or guardians. If the need for consent was waived by the ethics committee, please include this information. | This study used data from patient records (i.e., record of patients treated for MDR-TB at the study sites). In the context of the Oromia Region of Ethiopia, the regional  Public Health Emergency management and Health Research core-process is mandated to own health information (including patient data) and allow access for researchers. At health facilities (hospitals) patient data is safeguard by care providers. Legal access to facility level patient data is based on the ethical clearance written from the regional Public Health Emergency management and Health Research core-process, *that I have uploaded as an additional supporting document*. Based on the ethical clearance certificate from the legally mandated structure I was permitted by the respective hospital management and the caregivers there to access patient data. No data on personal identifier was collected to make sure that data remain anonymous.  *Note: Ethiopia uses its own calendar year which is some 9 years behind the Gregorian Calendar (I am including this to avoid confusion). The date on Ethical Clearance (2008 Ethiopian Calendar is written in Early 2017 GC.* |
| Development of the questionnaire for the study | At the conceptual phase selecting the topic for research was emanated from the researcher’s passionate interest to investigate factors that might influence treatment process and the outcome of treatment for MDR-TB. Such an interest was rooted in the researcher’s professional experience of the clinical and programmatic challenges associated with the management of MDR-TB. As a result, the research question of this study was informed by the researcher’s real-life experience on MDR-TB, which is a priority subject in Ethiopia.  The questionnaire was developed using the extant literature, and opinions of research experts and experts in MDR-TB, who conducted a serial review of the questionnaire before it was subjected to a preliminary investigation. The questionnaire was tested in the field and was further refined based on the outcomes before its use on the main study. Here I have attached the questionnaire used for this study. |
| Discrepancies in the number of patients with HIV positive result between table 1 and table 3 | Yes, the number of HIV positive patents was 34 in table 1 (n=136). Table 3 talks of those patients for whom final treatment outcome was determined (n=110). Yes, in table 3 27 (25%) are positive for HIV. The difference is because the denominator is not the same. |
| Clarity on the duration of the study | 26 December 2012 and 17 September 2016 was the time during which total of 182 patients with MDR-TB were enrolled for treatment at the two sites.  The study collected secondary data, i.e. patient data; and data was collected cross-sectionally from 10 November 2016 and 7 February 2017 on patients who fulfilled the inclusion criteria of the study. Thus, the two-time period were stated to mean two different things: 26 December 2012 and 17 September 2016 is the time during which the potential sample population was enrolled to treatment  2) 10 November 2016 and 7 February 2017 is the time during which data was collected for the study |
| HIV/AIDS written twice in table 1 | The first mention was on the proportion of patients MDR-TB and HIV co-infection and the second mention to disaggregate the contribution of HIV infection to total co-morbidity with MDR-TB among the study participants. Thus, I wanted to bold state HIV as major contributor to co-morbidity among patients with MDR-TB so that it gets the attention of readers (especially health policy makers in Ethiopia) as currently HIV co-infected patients are not getting services for both diseases under one roof and by the same caregiver! |
| Missing reference page | This is rephrased and corrected in the body of the manuscript. Thank you for demanding clarity in the review as it will be a concern for potential readers! |
| Recommendation to include newer data till December 2020 | Frankly speaking to one of my key research community, this research was part of a bigger research conducted for Doctoral programme at the University of South Africa. As you all now academic processes are very lengthy and time taking till decisions are made. Although it is in the heartfelt interest of the author to have more updated data, going back to the field is unachievable because of time and financial/logistical) constraints. I also believe that the editors understand the long journey a passionate researcher should go in sub-Saharan Africa to get to this point!! |
| Discrepancies in the number of patients with co-morbidity (HIV plus others) between table 1 and table 3 | Yes, in the same way for number of patients with positive HIV result the difference was because the denominator is not the same (41 patients had some co-morbidity with MDR-TB out of the total n=136; and 34 (31%) of the n=110 patients for whom final treatment result was assessed had some co-morbidity with MDR-TB. |
| The association between malnutrition and adverse drug reactions | As cited by the WHO (2013b:8), cited that malnutrition, as a co-morbid condition, is an indication of disease severity and poor patient response to treatment. In addition, it is associated with gastro-intestinal disorders and mal-absorption during treatment for MDR-TB  Caminero (2010:624), for example, cited that the use of fluoroquinolones in patients with low body weight is associated with more adverse drug reactions among patients treated for MDR-TB. |
| The study design is not clear. In one sentence it is written-‘both deductive and inductive approaches were used’. However, in the next sentence it is written- ‘Only deductive approach was used’. This needs clarification. | This research was part of a bigger research conducted for Doctoral programme at the University of South Africa. The entire research used both quantitative and qualitative approach to answer different problems of the same underlying research question. Yet, the two approaches were employed independently. Thus, we mentioned that infact the bigger study comprised of quantitative and qualitative components. This time I have understood the ambiguity that such phrases can create in the minds of the readers and omitted the phrase “mixed method” and adopted the ‘the quantitative approach’,- a design which is underpins this particular study. |
| There is non-uniformity in writing numerical values-at some place in text, it is written as digits and at some places as words. | This is corrected during current manuscript revision. Thank you for your prompt observation and comments!!! |
| What drug regimen was provided to MDR-TB patients? Is it the same regimen prescribed to all patients, or it is different for pulmonary and extrapulmonary cases and also in the same group? If different regimes are given to different patients, then it would be a major confounding factor in analyzing the disease outcome. | The study revealed that all patients (n=136) were treated using the WHO recommended standardized long treatment regimen for MDR-TB which is 8 (Z-Cm6-Lfx–Pto (Eto)–Cs for the intensive phase and 12 (Z-Lfx–Pto (Eto)–Cs for the continuation phase. Thus, all patients were enrolled to the same MDR-TB treatment regimen irrespective of their age or the type of TB as pulmonary or extra pulmonary.  Thus, there hasn’t been concerns that can arise if patients were enrolled to differing MDR-TB treatment regimens. |
| Does the patient need to pay for the treatment of TB or the drugs are provided free of cost by government of Ethiopia? | In policy and practice the patients get MDR-TB diagnosis and treatment services for free including the drugs used to treat the disease. Yet, patients and their families incur indirect catastrophic costs in the form of lost jobs due to the disease and the time needed to complete its treatment. Moreover, patients incur unnoticeable costs for transportation and accommodations while out of home. |
| The reference for classification of initial bacillary load as ‘scanty, moderate or high’ is required. It needs clear objective definition. | It has implications on the treatment outcome of patients with MDR-TB (Vishakha & Sanjay 2013 [47].)  The WHO criteria for estimating patient bacillary load at diagnosis is given as follows:   1) **No AFB** (Negative)=0 AFB /100 HPF (high power field) 2) **Scanty** (1+) =1-9 AFB/ 100 HPF 3) **Moderate** (2+) =10-99 AFB/100HPF 4) **High** (3+) = (1-10 AFB/1HPF/ 5) **Very High** (4+)/>10 AFB/1 HPF/   If TB is pulmonary and diagnostic sputum was done, semi-quantitative bacillary load is reported by laboratories at diagnosis. This helps to estimate initial bacillary load which has patient response implications during follow ups and patient education to halt further transmission of the disease to close contacts. |
| Which drug was responsible for suicide due to clinical psychiatric problem as an adverse drug reaction? How was the causal relationship established? | From second line drugs currently in use Cycloserine, is the most common cause of psychiatric disturbance like depression and psychosis. As one of the severe adverse drug reactions from drugs used to treat MDR-TB, psychiatric disorders are important barriers to patients’ adherence to treatment including patients committing suicidal acts like what is observed in this particular study. This problem can be anticipated in patients taking the drugs and, if routine clinical follow ups are provided appropriately, it can be picked early in the course of its development and managed by the attending team of caregivers. |
| The analysis should also be done comparing urban and rural population and its correlation with disease outcome. | Although this is logical observation and comment; yet getting adequate sample for the comparable groups needs inclusion of more patients in multiple treatment centers with huge resource implications. Although this has been in the heart of the researchers, the resources we had limited us from reaching more treatment centers to make meaningful internal comparison as rural vs urban. |
| There was no new information in the conclusion of the study. These are already established facts that malnutrition, poor socio-economic status, unemployment, co-infection with HIV and co-morbidities are responsible for poor disease outcome in MDR-TB cases. | For long time, Ethiopia has been one of the global high burden countries for TB, MDR-TB and TB/HIV. This study revealed that, there is high level of co-infection with MDR-TB and HIV/AIDS. Yet, there is no integrated care for MDR-TB and HIV/AIDS in the national program of MDR-TB (meaning that patients with MDR-TB and HIV/AIDS cannot get services for both diseases under one roof and by the same care provider). Patients are obliged to visit different care providers practicing in separate health facilities to get care for both diseases.  Moreover, despite the high prevalence of malnutrition among MDR-TB patients (as revealed in this study), there is no adequate socio-economic and nutrition support for the needy patients.  By uncovering the impact of malnutrition and the absence of integrated care for MDR-TB and associated co-morbidities, this study is in a better position to influence local policy makers towards establishment of integrated care on MDR-TB and HIV/AIDS and in addressing malnutrition and other socioeconomic constraints among patients treated for MDR-TB in Ethiopia. Caminero (2013: 201), cited that free nutrition intervention for MDR-TB patients should not be considered as an incentive but should rather be seen as a necessary intervention to facilitate treatment success as nutrition intervention has been proved to improves the body’s response to treatment and increases chances of patient survival. |
| Include additional information regarding the survey questionnaire | This part is amended by including details on the procedures followed in the development and use of the questionnaire. Found in the revised manuscript. |
| upload your figure files to the Preflight Analysis and Conversion Engine (PACE) digital diagnostic tool | This is done and the original tables are replaced by the one generated from the PACE. |
| Details of professional editor who edited the document | Attached as supporting document |
